# Observations on the Use of Nitric Acid in Hepatitis, in a Letter to Dr. Coxe

**Published:** 1807-10

**Authors:** S. Ffirth


### *Observations on the Use of Nitric Acid in Hepatitis, in a Letter to Dr. Coxe.* By S. Ffirth, M. D.

Sir,

That hepatitis is a frequent and troublesome disease in the East-Indies, is a fact long known; that mercury is almost a specific for the cure thereof, is also equally well known; but practitioners are fully aware that cases have, do, and may again occur, in which it would be improper to use this "Sampson of the materia medica;" and that, owing to the prejudices of patients, it cannot sometimes be administered, when no other impediment to its use exists. It becomes therefore of consequence to be acquainted with other articles that may be equally efficacious to exhibit in these cases. Of the numerous articles which have been tried, none have evinced such powers as the nitric acid, and none performed such wonderful cures. The attention of medical men were called to this subject some years ago, by Dr. Scott, an eminent practitioner at Bombay; but I do not think that his representations met with the attention which they so deservedly merited. I am convinced good nitric acid is one of the most valuable articles of the materia medica, and may be used with great advantage in *almost every* disease in which *mercury* is now given; and owing to its great tonic powers, it can be used in cases where the debility is so great as to forbid the use of the latter; and in hot climates, where great debility attends every disease, this virtue renders it the most valuable article the physician can prescribe. In cases of hepatitis, where the patient has a scorbutic habit of body, (and these cases are frequent amongst seamen in long voyages) the nitric acid can alone be used with success. It should be of the best kind, as I have found the common aqua fortis of the shops not to answer so well on several accounts, and it always contains a portion of muriatic acid, which is injurious in these cases. At first it should be given in small doses, frequently repeated, and largely diluted with water, and the dose gradually enlarged, as circumstances may indicate the necssity thereof. After continuing the use of it for some time, the mouth becomes affected, the salivary glands enlarge, their secretion is increased, the gums swell, become sore, and a disagreeable taste is complained of, a slight degree of fætor is produced, but very different from that which is occasioned by the preparations of mercury—the pain of the the side abates, and gradually becomes less troublesome, until it is felt no more, the pain in the shoulder subsides, the enlargement of the liver, which had previously taken place, ceases to proceed; it soon decreases to its natural size, and with it the nausea, oppression, and all the other disagreeable symptoms; the patient improves in health and strength continually, and from being debilitated, infirm, and wretched, becomes healthy, vigorous, and cheerful. The ptyalism produced is not so disagreeable as that arising from mercury, and is never followed by pains in the limbs; neither does it induce a predisposition to rheumatism, as many contend mercury does. It at the same time cures the scorbutic habit the patient had contracted; but if the scurvy had broken out, and made much progress, it will not effect a cure: the most that can be expected in these cases is, the cure of the hepatitis, and the preventing the farther advance of the other symptoms, which can alone be cured by limes, lemons, and oranges, or by living on fresh provisions. But, certainly, to cure hepatitis in a scorbutic patient by nitric acid, is doing a great deal; mercury, in such a case, would only increase the symptoms, and hasten the fatal termination thereof. I have used the nitric acid with the happiest success in a number of cases, one of which I will relate.

William Woods, a seaman of the China, had an attack of the malignant fever soon after the ship left Batavia, of which he recovered, but continued very weak and feeble for a considerable length of time. During the passage between Balambuan and St. Helena, he complained of pain in his side, pain in the right shoulder, sometimes in the clavicle, sometimes in the scapula; he felt a sense of weight, and "dragging," as he expressed it, whenever he lay on his left side. Upon examination I found the liver *very much enlarged,* occupying a large part of the abdomen, and painful when pressed. The man was much debilitated, had night sweats, very feeble and quick pulse, flushed cheeks, or rather a circumscribed spot on each cheek, such as usually is seen in hectic fever; he complained of pains in his limbs, had a scorbutic eruption on his lower extremities, with purple spots on some parts of his body. I thought the case desperate, but determined to do all in my power to give him relief; and, upon considering his case maturely, thought nothing promised so fair to do this as the nitric acid. I accordingly prescribed a drachm of it to be diluted in a quart of water, and taken in the course of the day; the second day he took a drachm and and a half in three pints of water; the third day, two drachms, as he had borne the first increase of the medicine very well. It was very grateful to his taste, and allayed his thirst. I continued at two drachms for four days, when it was increased to two and a half, diluted with three quarts of water, and taken for his constant drink through the day and night. In this manner the dose was increased to half an ounce in the course of three weeks, beyond which I did not increase it. His strength increased, his appetite returned, his scorbutic habit was almost gone, the medicine began to affect the gums, and at the end of the fourth week the pain in the shonlder had greatly diminished, and was not troublesome; the liver appeared to be decreased in size: he was in good spirits, spat freely, and said he could lie on his left side without experiencing so much uneasiness. From this time the diseased liver gradually diminished in size, his health and strength returned, and after continuing the medicine nine weeks, he was discharged, perfectly cured.

I could add several other cases in which it was equally efficacious in curing the disease, but it is unecessary; suffice it to say, that I have never failed with it, although I have no doubt that cases may occur in which it will not succeed, particularly where *very extensive suppuration* has already taken place, or where suppuration, combined with *scurvy*, in a very considerable degree, exists; but in these cases no medicines will effect a cure at sea; and even if the patient is on shore, and has fresh animal and vegetable food, his chance of recovery is *very small.*

With respect to the modus operandi of nitric acid, I must say I am doubtful. It is a tonic, every person must admit; that it is a stimulant, none can deny. It salivates; so in some instances does copper and oxygenated muriatic acid: so does the best acetic acid; so will opium, cicuta, hyosciamus niger, &c. when given to a certain extent. In this they all *appear to approach somewhat* towards the nature of mercury; yet every one admits them to be very different. Acids contain oxygen, mercury is only active in proportion to the quantity thereof that exists in its different preparations. But do opium and hemlock also contain oxygen? If not, does salivation depend upon oxygen in certain cases, and not in others? It certainly must depend upon *something* in common to all articles that salivate. We know that seneka polygala, and several other powerful stimuli, will produce ptyalism. No person will contend, contend, that seneka contains oxygen; but if we view the action of all these medicines, we shall find they have one thing in common, that is, they are all powerful incitants; but it may be asked, if ptyalism depends upon an incitant action, why do not all articles of that class salivate? Perhaps it may be owing to a *peculiar* stimulating power possessed by these articles, and not by the others. What is this peculiar power? I do not know; that it is *not* oxygen, I believe; but what it is I cannot pretend to say. There are many things which we know, but cannot explain. What is matter?—What is excitability?—What is life? We know all these to be; but where is there a philosopher can say in what they consist? So in like manner with the salivating principle: we know it is something: we know it must be possessed in common by several articles *apparently very different*; but what it is, as I have said in the beginning of this discussion, *I am uncertain.* I hope some of your learned correspondents will solve my doubts. With this view I submit them to the public.

